# Supplementary material for: Increased circulating TREM2+ microglial extracellular vesicles in aged APP/PS1 Alzheimer's disease rats
Source: Brain Behav Immun Health. 2026 May 12;54:101261. doi: 10.1016/j.bbih.2026.101261 (PMC13195343; doi:10.1016/j.bbih.2026.101261)

**Supplemental Information**

**Increased circulating TREM2^+^ microglial extracellular vesicles in aged APP/PS1 Alzheimer’s disease rats**

Sarah J. Myers^1^, Manoj Reddy Medapati^1^, Brian L. Allman^1^, Stephen H. Pasternak^2,3^, Shawn N. Whitehead^1,*^, Austyn D. Roseborough^1,*^

^1^Vulnerable Brain Lab, Department of Anatomy and Cell Biology, Schulich School of Medicine and Dentistry, Western University, London, Ontario, Canada

^2^Department of Clinical Neurological Sciences, Schulich School of Medicine and Dentistry, Western University, London, Ontario, Canada

^3^Robarts Research Institute, Schulich School of Medicine and Dentistry, Western University, London, Ontario, Canada

^*^Indicates authors contributed equally and share senior authorship

**Table S1: Primer sequences used for qPCR experiments**

| **Gene** | **Forward Primer** | **Reverse Primer** |
| --- | --- | --- |
| Rpl13⍺ | GCTGTGAGGGCATCAACATT | TTGGTGTTCATCCGCTTTCG |
| β-actin | GGCACCACACCTTCTACAATG | GGGGTGTTGAAGGTCTCAAAC |
| TMEM119 | TCCTTTTCCCAGAGCTGGTTC | GAAGAACCCTCAGCTTCCCC |
| TREM2 | AACTTCAGATCCTCACTGGACC | ATTGGATTGGCTCCTGGCTG |

**Figure S1: Nanoflow detection of TMEM119, TREM2, and standardized beads in rat plasma.** A-B) Scatter plots of silicon beads (180 nm, 240 nm, 300 nm, 590 nm, and 880 nm) and 488-conjugated polystyrene beads (100 nm and 500 nm). C) Scatter plot of total plasma EVs. D-F) PBS in 647-, 405, and dual-gated channels. G-I) Plasma in 647-, 405-, and dual-gated channels. J-L) Plasma + TMEM119-647 antibody in 647-, 405-, and dual-gated channels. M-O) Plasma + TREM2-405 in 647-, 405-, and dual-gated channels. LALS, long angle light scatter; SALS, short angle light scatter.

**
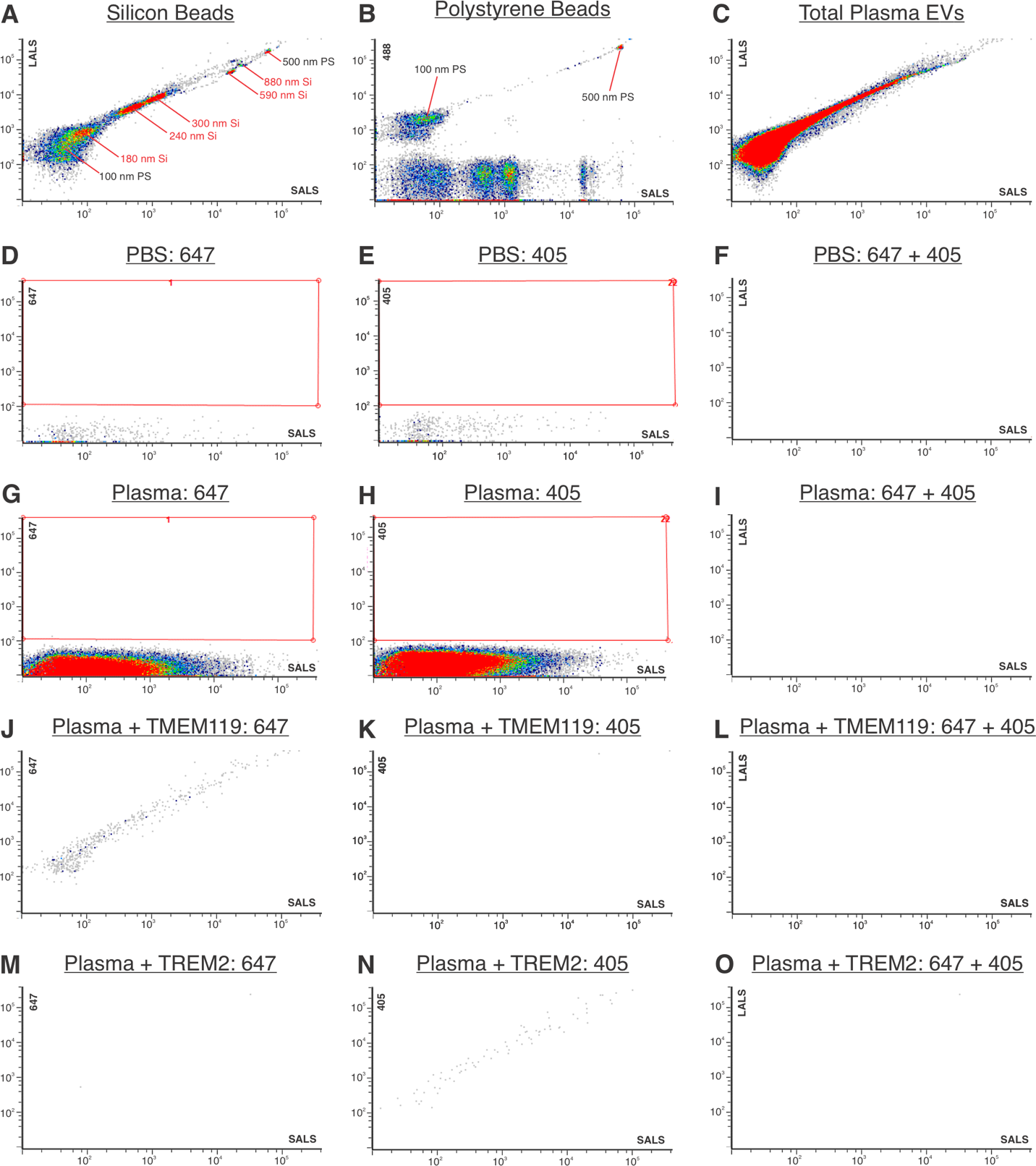
**

**Figure S2: Hippocampus western blot imaging files**

**
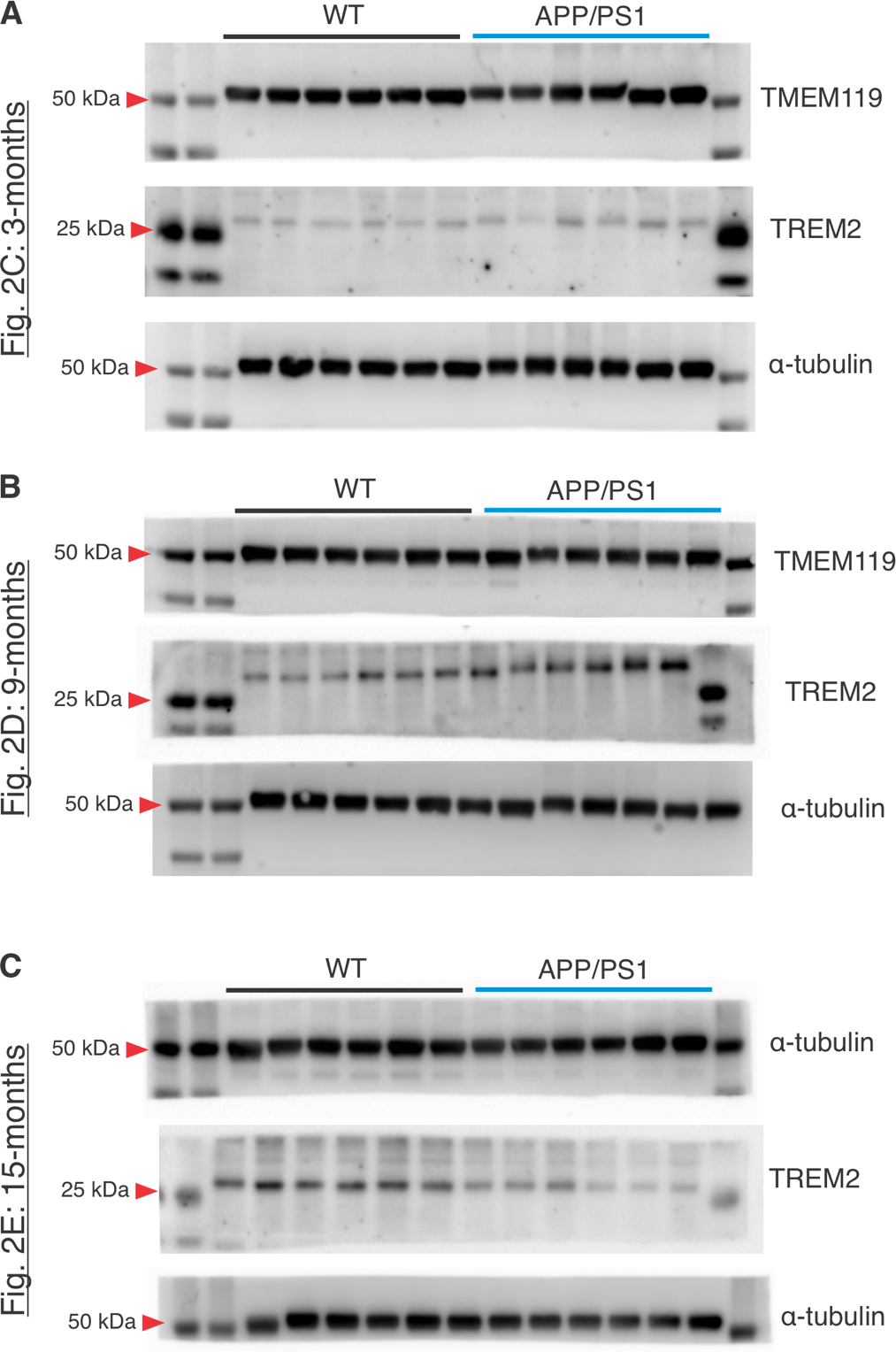
**

**Figure S3: Corpus callosum western blot imaging files**

**
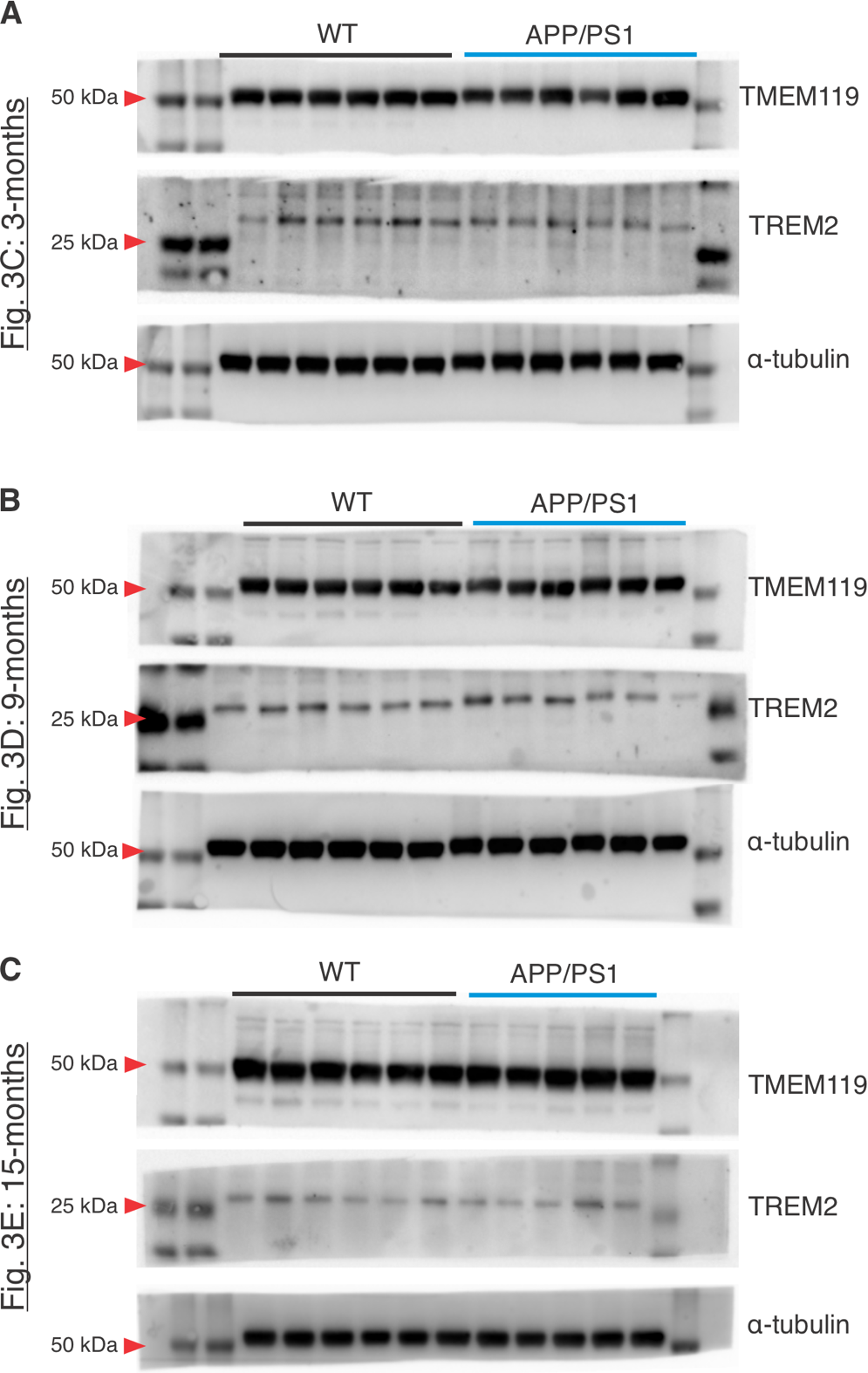
**

**Figure S4: EV isolation western blot imaging files**

**
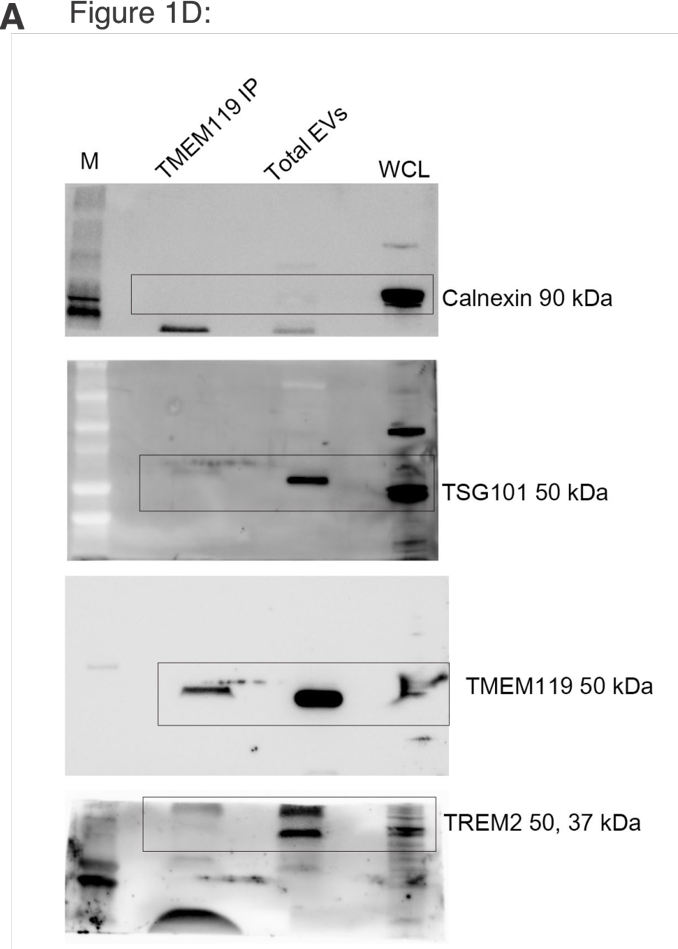
**

**Figure S5: No differences in single-positive TMEM119 or TREM2 EV populations.** A) Nanoscale flow cytometry detection of TMEM119^+^ events/µl in plasma from 3-, 9-, and 15-month-old wildtype and APP/PS1 rats and assessed using a 2-way ANOVA (genotype ✕ sex). B) Nanoscale flow cytometry detection of TREM2^+^ events/µl in plasma from 3-, 9-, and 15-month-old wildtype and APP/PS1 rats and assessed using a 2-way ANOVA (genotype ✕ sex). Data represent the group mean ± SEM. *n* = 10-12 males and females per group.


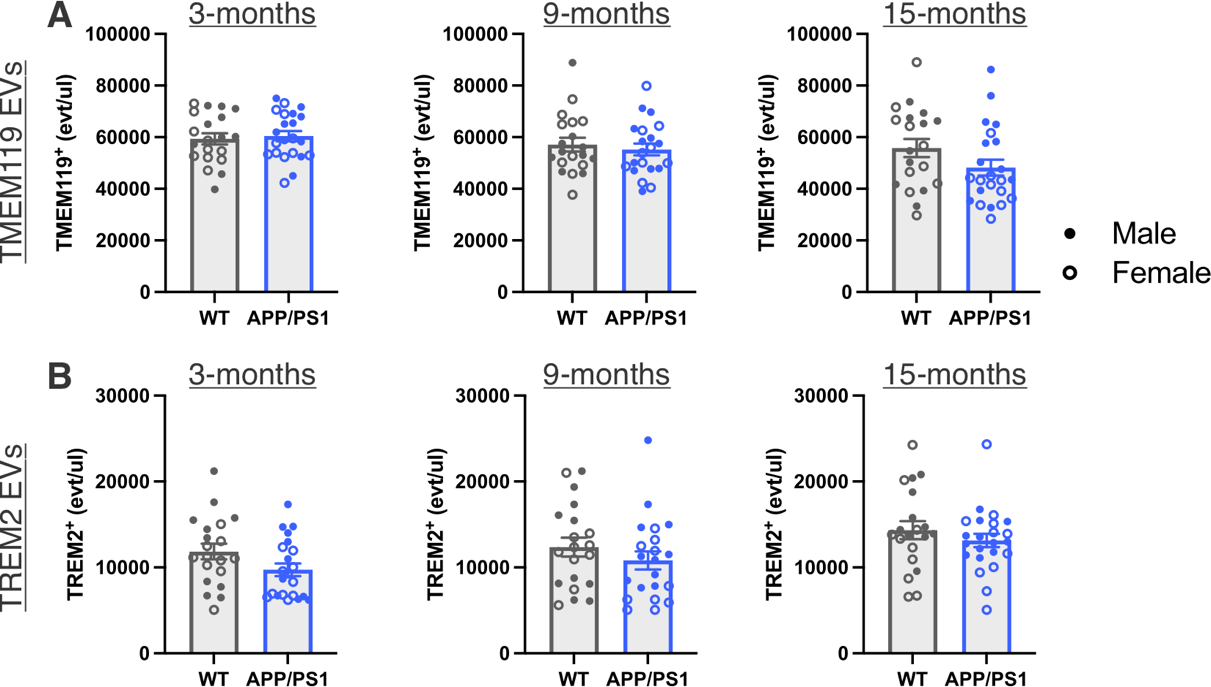


**Figure S6: Discrimination of wildtype from APP/PS1 rats using TMEM119^+^/TREM2^+^ EVs.** Receiver operating characteristic (ROC) curves of 3-, 9-, and 15-month rats and area under the curve (AUC) values.

**
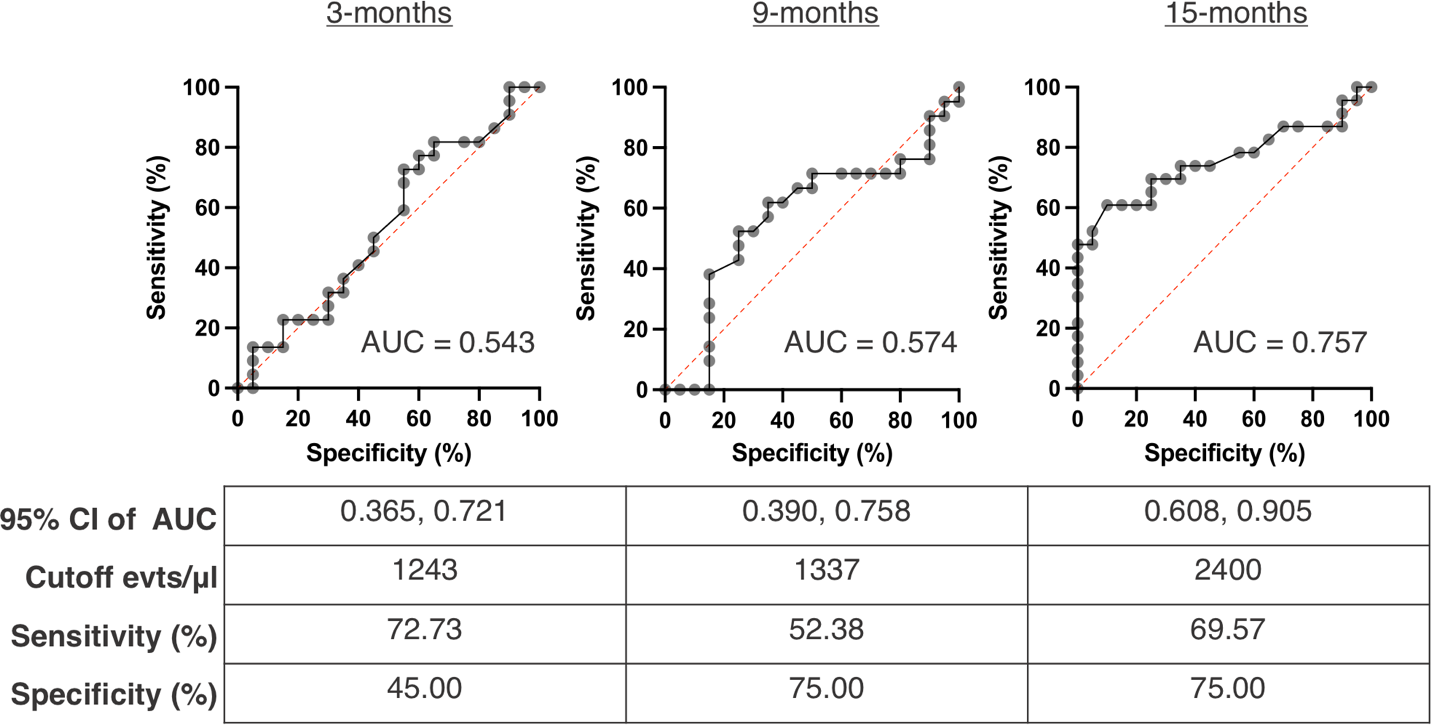
**

**Figure S7: Immunohistochemistry of 82E1 for amyloid deposition** **in 3-, 9-, and 15-month APP/PS1 rats.** A-C) Left panel images are 10 ✕ stitches of the hippocampus (scale bar indicates 500 µm) and right panel images are 20 ✕ captures of the outlined area (scale bar indicates 50 µm) in A) 3-month, B), 9-month, and C) 15-month APP/PS1 rats. D) Image taken at 40 ✕ magnification depicting amyloid plaque (scale bar indicates 25 µm).

**
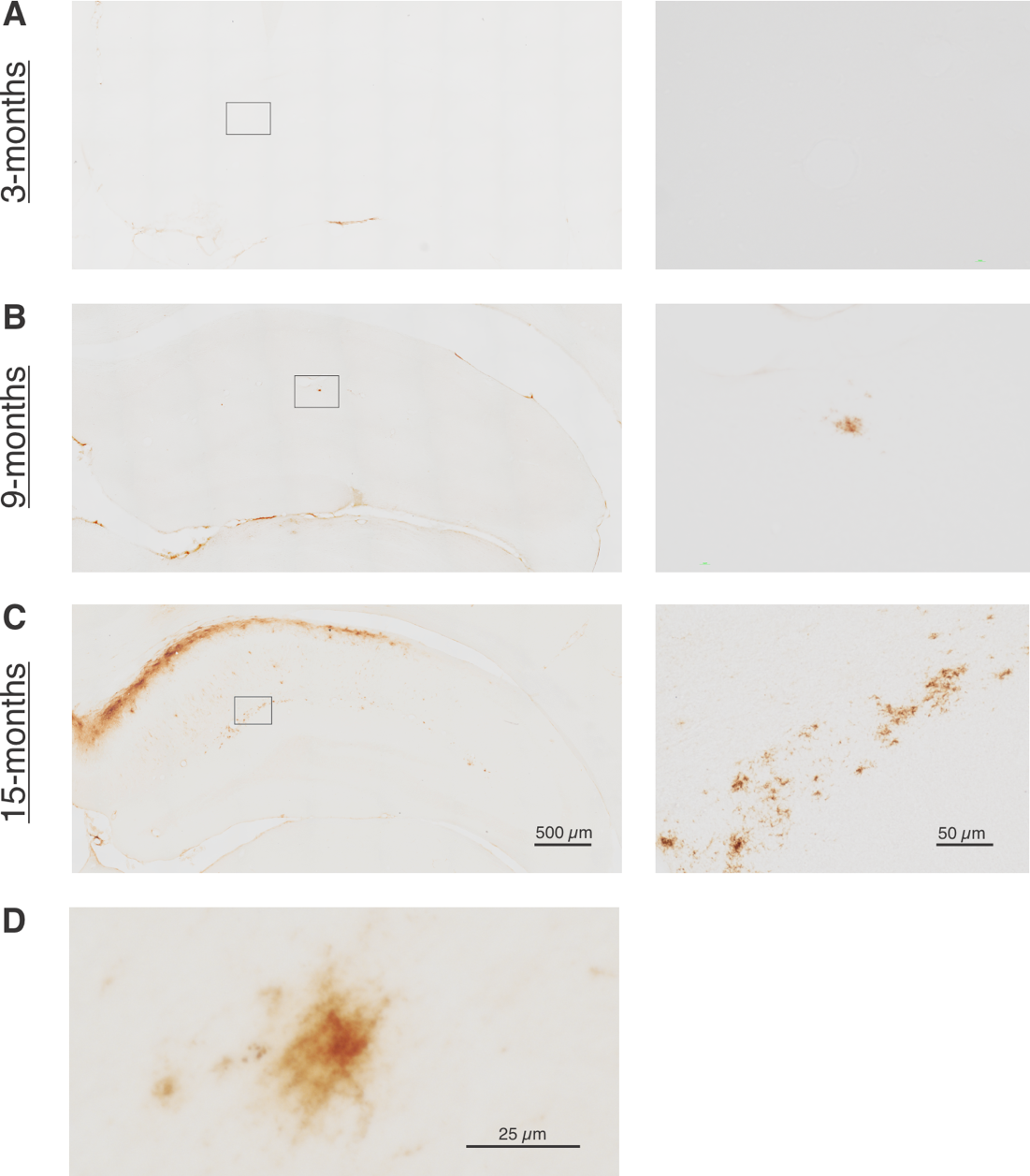
**

**Figure S8: Visually guided trials confirm no visual acuity deficits across groups.** A) Rats underwent 4 trials in which a platform was visually marked with a flag. Average time taken to reach the platform across the 4 trials was assessed in 3-, 9-, and 15-month-old wildtype and APP/PS1 rats using a 2-way ANOVA (genotype ✕ sex). B) Swim speed was averaged across the 4 visually guided trials and assessed using a 2-way ANOVA (genotype ✕ sex). * Indicates statistical significance (*p* < 0.05). Data represent the group mean ± SEM. *n* = 10-12 males and females per group.


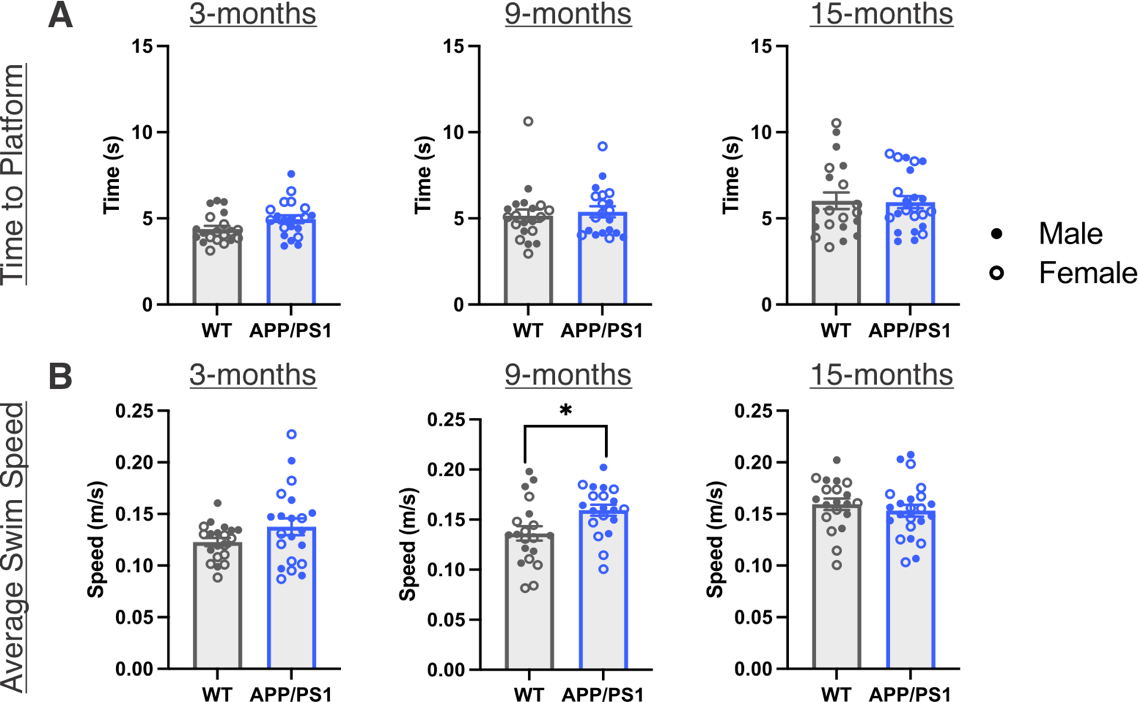

Supplement: Multimedia component 1 [file mmc1.docx]
